# Incidental detection of an occult oral malignancy with autofluorescence imaging: a case report

**DOI:** 10.1186/1758-3284-1-37

**Published:** 2009-10-28

**Authors:** Nadarajah Vigneswaran, Sheila Koh, Ann Gillenwater

**Affiliations:** 1 Department of Diagnostic Sciences, The University of Texas Dental Branch at Houston, Houston, Texas 77030, USA; 2 Department of Restorative Dentistry and Biomaterials, The University of Texas Dental Branch at Houston, Houston, Texas 77030, USA; 3 Department of Head and Neck Surgery, University of Texas M.D. Anderson Cancer Center, Houston, Texas 77030, USA

## Abstract

**Background:**

Autofluorescence imaging is used widely for diagnostic evaluation of various epithelial malignancies. Cancerous lesions display loss of autofluorescence due to malignant changes in epithelium and subepithelial stroma. Carcinoma of unknown primary site presents with lymph node or distant metastasis, for which the site of primary tumour is not detectable. We describe here the use of autofluorescence imaging for detecting a clinically innocuous appearing occult malignancy of the palate which upon pathological examination was consistent with a metastatic squamous cell carcinoma.

**Case Description:**

A submucosal nodule was noted on the right posterior hard palate of a 59-year-old white female during clinical examination. Examination of this lesion using a multispectral oral cancer screening device revealed loss of autofluorescence at 405 nm illumination. An excisional biopsy of this nodule, confirmed the presence of a metastatic squamous cell carcinoma. Four years ago, this patient was diagnosed with metastatic squamous cell carcinoma of the right mid-jugular lymph node of unknown primary. She was treated with external beam irradiation and remained disease free until current presentation.

**Conclusion:**

This case illustrates the important role played by autofluorescence tissue imaging in diagnosing a metastatic palatal tumour that appeared clinically innocuous and otherwise would not have been biopsied.

## Background

Light-induced tissue autofluorescence examination is currently considered as a standard of care for screening and diagnostic evaluation of early neoplastic changes of the skin, cervix, lung, bladder, gastrointestinal tract and oral cavity [1-11]. Dysplastic and cancerous tissues often exhibit decreased blue-green autofluorescence and appear darker compared to uninvolved mucosa. Most of this reduction in perceived fluorescence is attributed to diminished signal (detectable from the surface) that emanates from collagen crosslink within the subepithelial stroma [12].

Recently the U.S. Food and Drug Administration has approved autofluorescence-based oral mucosal screening devises which are marketed as VELscope and Identafi™ 3000 for early detection of potentially malignant oral lesions. Here we report of a case to highlight the value of tissue autofluorescence visualization in diagnosing a squamous cell carcinoma, metastatic to the palate, which clinically presented as an innocuous appearing submucosal nodule.

## Case Report

A 59-year-old white female was receiving regular dental treatment at the Special Patient Clinic of the University of Texas Dental Branch at Houston. During her recent clinic visit, we noticed a submucosal nodule on her posterior hard palate during routine oral examination. The patient was unaware of this lesion. This lesion was covered by intact mucosa, was non-fluctuant and had the consistency of an irritation fibroma. This submucosal nodule measured 0.8 cm in greatest diameter and palpation of this nodule elicited no tenderness or blanching.

Her medical history is significant for cervical lymph node metastasis of squamous cell carcinoma from unknown primary, hypertension, chronic obstructive pulmonary disease, hypothyroidism and depression. The patient was diagnosed with squamous cell carcinoma of unknown primary, metastatic to the right mid jugular lymph nodes approximately four years ago. Various imaging studies were performed at the time which failed to identify the primary site of this tumour. The patient was treated with a course of external beam radiation therapy to the pharyngeal axis and the neck. Her oral mucosa and teeth were shielded from the direct radiation therapy beams. Patient had no clinical evidence of either primary or metastatic tumour during the last four years. Her current medications include levothyroxine, enalapril malate and fluoxetine. The patient had a 10-pack-year history of smoking and social consumption of alcohol. The patient's family history was unremarkable.

**Figure 1:**
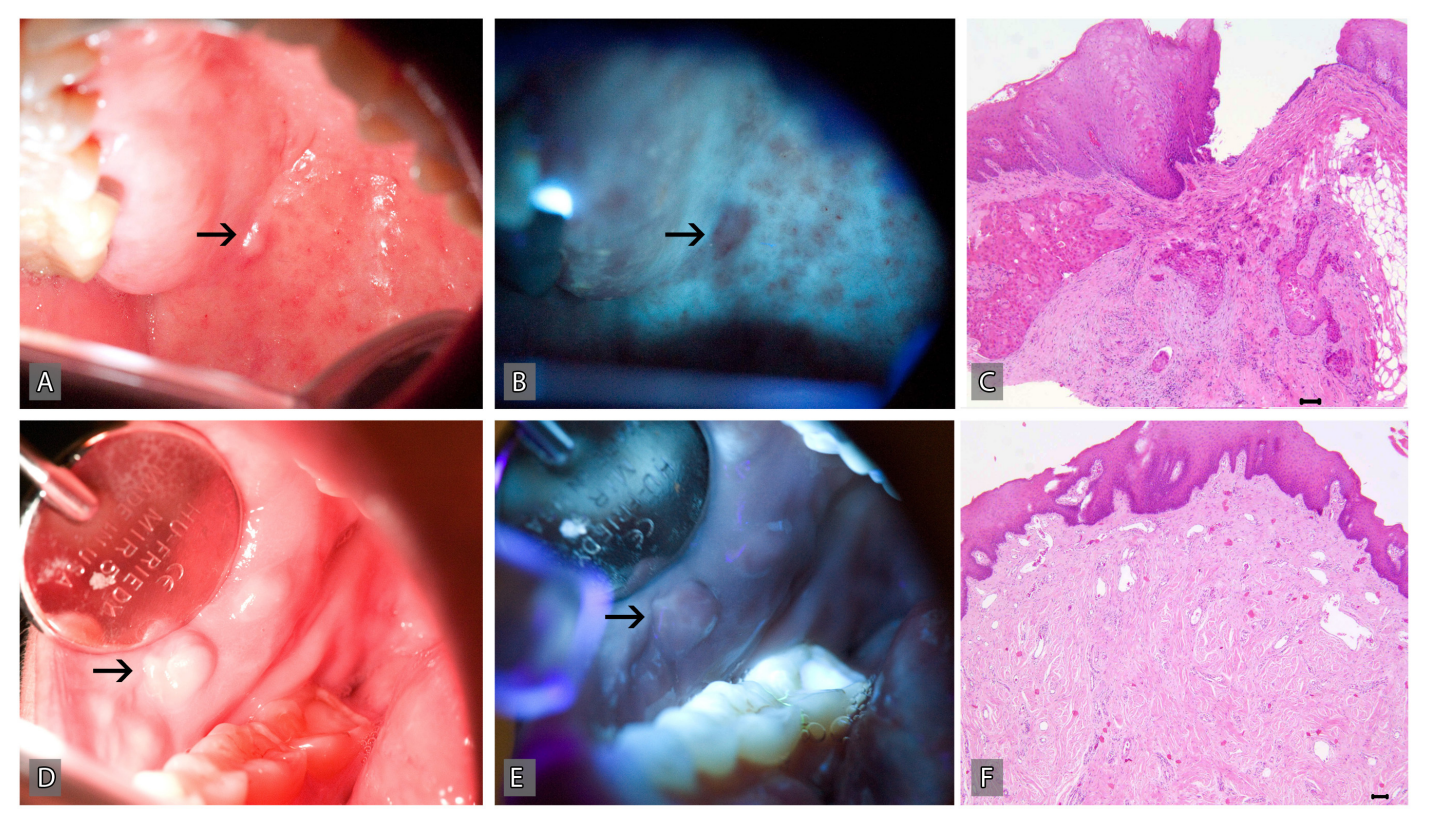
**An innocuous appearing submucosal nodule (arrow) was noted on the posterior hard palate of a 59-year-old white female during standard clinical examination which upon examination with a multispectral oral cancer screening device (Identafi™ 3000), revealed loss of autofluorescence**. A: White light image of the metastatic lesion; B: Fluorescence image at 405 nm illumination; C: Microscopic examination of the biopsy revealed the presence of metastatic squamous carcinoma, Bar = 100 μM (C). In comparison, a similar appearing benign fibroma (arrow) exhibits normal autofluorescence. D: White light image of a benign fibroma; E: Fluorescence image at 405 nm illumination; F: Microscopic examination of the biopsy revealed reactive fibrous hyperplasia, Bar = 100 μM.

Our clinical differential diagnosis for this palatal nodule included traumatic fibroma and epidermoid cyst. However, because of her past medical history of metastatic disease, we examined this nodule using a multispectral oral cancer screening device (Identafi™ 3000). This submucosal nodule revealed decreased autofluorescence at 405 nm illumination (Figure 1A-C). On the other hand, a clinically similar appearing benign fibroma (arrow) from a different patient, exhibited normal tissue autofluorescence (Figure 1D-F). An excisional biopsy of this submucosal nodule was performed which was diagnosed as consistent with metastatic squamous cell carcinoma because of the overlying benign surface epithelium. Patient was referred to her oncologist for further evaluation and treatment. The clinical protocol for the detection potentially malignant oral mucosal lesions with Identafi 3000 was reviewed and approved by the Institutional Review Board of the University of Texas Health Science Center at Houston. Written informed consents were obtained from all participants of this clinical study.

## Discussion

Increasing need for additional non-invasive tests to augment conventional white light oral examination has driven the development of various real-time light-induced fluorescence visualization devices for detection and delineation of potentially malignant oral lesions [9,13]. The U.S. Food and Drug administration has approved two oral cancer screening devices namely, VELscope^® ^(LED Dental, Inc, White Rock, British Columbia, Canada) and Identafi 3000™ (Trimira™, Houston, Texas) for light-induced fluorescence visualization of oral mucosa. VELscope uses a blue/violet light (400-460 nm) to illuminate oral mucosa and a specific filter allows the clinician to visualize tissue autofluorescence. Identafi 3000 uses multiple illumination settings namely white, amber (560 nm) and violet (405 nm) to visualize the oral mucosal reflectance (white and amber) and fluorescence (violet).

Light-induced tissue fluorescence visualization technologies are being used increasingly as non-invasive diagnostic aids to characterize biochemical and structural changes associated with neoplastic transformation [7,11,14]. These diagnostic tests vary depending on the type of fluorophores being visualized: 1) endogenous fluorophores (autofluorescence); 2) fluorophores synthesized in tissue after administration of a precursor drug; 3) fluorophores injected as exogenous drug. Autofluorescence visual imaging is the most widely used and safest method that has great promise to enhance the visualization and diagnostic predictability of potentially malignant lesions. When normal mucosa is illuminated by high-intensity violet (405 nm with the Identafi™ 3000 system) or blue light (436 nm with VELscope system), specific components of this mucosa (fluorophores) emit low-energy light, which is visualized as the autofluorescence image of the mucosa. The major fluorophores of oral mucosa include flavin adenine dinucleotide (FAD) and nicotinamide adenine dinucleotide (NADH) of the epithelium and cross-linked collagen and elastin fibers of the underlying lamina propria. Subepithelial stromal collagen fibers are the predominant source of autofluorescence in oral mucosa [15]. Usually malignant transformation is associated with thickening of the epithelium, enhanced cellular density due to increased nuclear cytoplasmic ratio which may attenuate the excitation leading to a decrease in collagen autofluorescence. Although the role of autofluorescence based screening devices such as the Identafi™ 3000 or the VELscope in oral precancer and cancer diagnosis remains to be determined, recent studies have documented promising applications for these devices. Recently, Roblyer et al. examined light-induced tissue autofluorescence at multiple excitation wave lengths at 365, 380, 405 and 450 nm and demonstrated that they could differentiate the dysplastic epithelia and carcinoma from histologically normal mucosa with 95.9% sensitivity and 96.2% specificity [16]. Moreover, subclinical premalignant and malignant lesions that are not visible on routine white light oral examination become noticeable with direct autofluorescence visualization [17]. Recent studies reported that autofluorescence tissue imaging is more sensitive than routine white light examination to determine surgical margins at the primary site that are free of histologic and molecular features of malignancy or dysplasia [18,19].

Our patient was diagnosed with carcinoma of unknown primary site (CUP), defined as metastatic carcinoma present in the lymph nodes with no identifiable primary tumour despite thorough clinical and radiographic evaluations [20]. The incidence of CUP, which represents up to 7% of all head and neck carcinomas, has reduced in recent years due to the use of multimodal imaging studies to identify occult primary tumours [21-24]. Approximately 75% of CUP occurring in the cervical lymph nodes in which the primary tumours were subsequently identified originated from the head and neck area, most frequently from the peritonsillar area and base of the tongue [25]. However, in a significant proportion of CUP patients, the primary tumour site may remain undetected for several years as in the current case [24,26]. The patient in our case had no clinical evidence of primary tumour or recurrence for 4 years until she presented with the described palatal tumour. We believe that this tumour represents a second metastasis and not a primary tumour because the surface epithelium overlying this submucosal tumour was not directly connected to the tumour island and did not exhibit histologic features of dysplasia or malignancy as noted with primary oral squamous cell carcinomas. To our knowledge, this is the first case to report the use of tissue autofluorescence imaging to diagnose an occult metastatic squamous cell carcinoma to the palatal mucosa. In the current case, it appears that metastatic tumour islands within the subepithelial stroma disrupted stromal collagen fibers leading to the loss of stromal autofluorescence. In contrast, a clinically similar appearing benign fibroma comprised of dense subepithelial fibrous tissue revealed normal autofluorescence. Certainly, multispectral tissue autofluorescence is a new and powerful technology that can be used for multiple diagnostic applications.

## Consent

Written informed consent was obtained from the patient for publication of this case report and accompanying images. A copy of the written consent is available for review by the Editor-in-Chief of this journal.

## Competing interests

NV serve as an investigator in a clinical study evaluating the oral cancer screening devise "Identafi 3000™" which was sponsored by Trimira, Houston, Texas.

AG is a co-owner of patents that have been licensed to Remicalm LLC, Houston, Texas, Trimira's parent company and serves as an unpaid scientific advisor to Remicalm LLC.

## Authors' contributions

NV, SK and AG participated in the diagnostic evaluation and management of the patient. All authors read and approved the final manuscript
